# Global Burden of Early-Onset Ischemic Heart Disease, 1990 to 2019

**DOI:** 10.1016/j.jacadv.2024.101466

**Published:** 2024-12-20

**Authors:** Xiao Liu, Yuting Wu, Fei Li, Xinrui Qi, Liyan Niu, Yifan Wu, Jitao Ling, Wengen Zhu, Qingqing Li, Xinyu Liu, Jing Zhang, Yunfeng Shen, Zhiwei Yan, Deju Zhang, Jingfeng Wang, Yuling Zhang, Peng Yu

**Affiliations:** a Department of Cardiology, Sun Yat-sen Memorial Hospital of Sun Yat-sen University, Guangzhou, Guangdong, China; b Guangzhou Key Laboratory of Molecular Mechanism and Translation in Major Cardiovascular Disease, Guangdong Provincial Key Laboratory of Malignant Tumor Epigenetics and Gene Regulation, Guangdong-Hong Kong Joint Laboratory for RNA Medicine, Guangzhou, Guangdong, China; c Institute for the Study of Endocrinology and Metabolism in Jiangxi Province, The Second Affiliated Hospital of Nanchang University, Nanchang, China; d Department of Endocrinology Medicine, The Second Affiliated Hospital, Jiangxi Medical College, Nanchang University, Nanchang, Jiangxi, China; e Department of Endocrinology and Metabolism, The Fourth People's Hospital, Shenzhen, Guangdong, China; f Department of Cardiology, The First Affiliated Hospital of Sun Yat-sen University, Guangzhou, Guangdong, China; g Department of Biochemistry and Molecular Biology, Johns Hopkins Bloomberg School of Public Health, Baltimore, MD, USA; h Department of Anesthesiology, The Second Affiliated Hospital, Jiangxi Medical College, Nanchang University, Nanchang, Jiangxi, China; i Department of Endocrinology, The Eighth Affiliated Hospital of Sun Yat-sen University, Shenzhen, Guangdong, China; j School of Physical Education and Sport Science, Fujian Normal University, Fuzhou, China; k Food and Nutritional Sciences, School of Biological Sciences, The University of Hong Kong, Hong Kong, China

**Keywords:** cardiovascular diseases, death, global burden of disease, ischemic heart disease, disability-adjusted life years, incidence, prevalence

## Abstract

**Background:**

Early-onset ischemic heart disease (IHD) is a growing burden associated with high disability and death.

**Objectives:**

This study aimed to estimate the burden of incidence, prevalence, and disability-adjusted life years (DALY) of early-onset IHD from 1990 to 2019.

**Methods:**

Data on the burden of early-onset IHD (men<55 years, women<65 years), including prevalence, incidence, DALY, and deaths, were collected from the Global Burden of Disease study for 204 countries and territories from 1990 to 2019.

**Results:**

In 2019, early-onset IHD affected 5.34 million (95% uncertainty interval [UI]: 3.96-6.96) individuals globally. This resulted in 58.48 million (95% UI: 52.65-64.69) DALY and 1.44 million (95% UI: 1.29-1.59) deaths. Countries with a middle sociodemographic index (SDI) had the highest incidence cases (1.79 million, 95% UI: 1.32-2.34) and the low-middle SDI countries had the highest age-standardized incidence rate of 179.34 per 100,000 (95% UI: 134.38-231.94). Low-middle SDI countries exhibited the highest age-standardized death rate (57.13 per 100,000, 95% UI: 48.45-66.53) and age-standardized DALY rate (2,309.67 per 100,000, 95% UI: 1962.31-2,693.93). Globally, female incidence cases (2.77 million, 95% UI: 2.04-3.64) surpassed male (2.57 million, 95% UI: 1.92-3.32). The top 2 attributable risk factors were high low-density lipoprotein cholesterol and high systolic blood pressure.

**Conclusions:**

The global incidence rate of early-onset IHD decreased from 1990 to 2019. The incidence rate was highest in low-middle SDI countries, and the burden of DALY was highest in low-middle SDI countries. The DALY and death rate were higher in men. High low-density lipoprotein cholesterol and high systolic blood pressure were the primary attributors.

Ischemic heart disease (IHD), also known as coronary artery disease, is the leading cause of death globally and a major cause of disability.^1^, ^2^, ^3^, ^4^ IHD exacts a heavy burden on human life and well-being, with over 8.9 million-related deaths occurring in 2017 alone,^5^^,^^6^ reaffirming its status as the leading cause of mortality worldwide.^7^ In a study investigating coronary heart disease mortality among U.S. adults over the 30 years between 1979 and 2011, there was a steady decline in mortality among adults aged 65 years. However, there was little improvement in mortality among younger men and women (age <55 years) in the 2 decades after 1990 to 2011.^5^^,^^8^ Current screening guidelines underestimate the risk of death from early-onset IHD.^9^^,^^10^ Evidenced from a prospective multicenter cohort study of AFIJI (Appraisal of Risk Factors in Young Ischemic Patients Justifying Aggressive Intervention) showed 18.2% of patients who experienced IHD were under 35 years old after a 20-year follow-up. Early-onset IHD remains an Aggressive disease with high recurrence and mortality rates.^11^, ^12^, ^13^ Consequently, it is imperative to comprehend the worldwide impact of disease and the risk factors related to early-onset IHD.

Despite several excellent studies investigating the global burden of disease and IHD,^3^^,^^6^^,^^14^^,^^15^ research on long-term trends in early-onset IHD and country-specific differences in gender and socioeconomic development remains limited.^16^ This dearth of research slows down the formulation of comprehensive strategies to tackle this issue on a global scale, catering to varying regional and national contexts.^9^ Thus, our study aimed to focus on the global burden of early-onset IHD in men (<55 years old) and women (<65 years old) based on the 2019 Global Burden of Disease Study.

## Methods

### Data source

The dataset concerning the disease burden of early-onset IHD patients (men <55 years of age, women <65 years of age) was retrieved from the Global Health Data Exchange query tool^59^ from 204 countries and territories on the annual incidence, prevalence, disability-adjusted life years (DALY), and death. IHD was defined as acute myocardial infarction, chronic stable angina, chronic IHD, and heart failure due to IHD.^3^ However, incidence data for IHD were limited to myocardial infarction in the GBD 2019 study. The GBD study is a collaborative project involving researchers from more than 150 countries, led by the Institute for Health Metrics and Evaluation at the University of Washington. It is recognized for its comprehensive approach and is widely used by policymakers, researchers, and health organizations to inform health policy and resource allocation. This study utilized mortality rates and DALY per 100,000 people to assess the disease burden of IHD.

### Disability-adjusted life years and socio-demographic indices

In this analysis, we looked at the significance of the unit of measurement known as DALY, which measures disease burden by calculating the total number of healthy years lost from the onset of an illness until death. DALY takes into account 2 distinct components: the years of life lost due to premature death and the years of life lost due to disability caused by illness.^17^ Additionally, we examined sociodemographic indices (SDIs), a well-rounded index that assesses a country's developmental status based on an inclusive evaluation of average education level, total fertility rate, and per capita income.^18^ These factors are combined into an index between 0 and 1, with 0 representing the lowest level of development and 1 the highest. SDI stratification is usually based on the distribution of SDI values globally. According to GBD 2017, the SDI stratification is as follows: low SDI: SDI values below 0.46; low-middle SDI: SDI values between 0.46 and 0.60; middle SDI: SDI values between 0.61 and 0.69; high-middle SDI: SDI values between 0.70 and 0.81; high SDI: SDI value above 0.81. This information can be found on the official GBD website, and the GBD 2019 SDI 1950 to 2019 data set provided by the Institute for Health Metrics and Evaluation details SDI values for all estimated Global Burden of Disease 2019 locations, as well as location groupings based on 2019 values.^60^

### Statistical analysis

Age standardization allowed for meaningful comparisons across populations by adjusting for differences in age distribution, ensuring accurate assessments within the specified age groups. Data for men <55 years of age and women <65 years of age were analyzed. Age-standardized rates (per 100,000 people) for the prevalence of early-onset IHD, DALY, and deaths were calculated using the global standard population from the GBD 2019 study. The formula applied was $\frac{\sum_{i=1}^{A}a_{i}w_{i}}{\sum_{i=1}^{A}w_{i}}\times 100,000$, where $a_{i}$ represents age-specific rates, and $w_{i}$ is the weight of the selected reference standard population in the same age group i.^1^^,^^19^^,^^20^

The estimated annual percentage change (EAPC) describes the trend in the age-standardized rate for a specific time interval, and we calculated the EAPC to quantify the trend in each of the metrics of early-onset IHD, assuming that the natural logarithm of the age-standardized rate is linear concerning time, using the following equation, viz:

y = α+βx+ε

(y = ln (age-standard rate), x = calendar year, ε = error term)

A subsequent 95% CI can be obtained from the linear model. The unit of EAPC is the percentage (%), which indicates the average percentage change from year to year. Positive EAPC numbers indicate an increased trend; negative numbers indicate a decreased trend.^21^ Moreover, we determined the annual average percentage changes using joint point regression analysis to ascertain the direction and extent of temporal trends in early-onset IHD incidence, DALY, and deaths. Average annual percent change is a measure of trends over specific time intervals that provide a simple way to describe the change in mean annual percentage changes over time, calculated as weighted averages of annual percentage change. We also used joint-point regression to analyze the epidemiological characteristics of IHD. The joint-point regression model is a set of nonlinear statistical models that are computed by estimating the pattern of change in prevalence by least squares, avoiding the lack of objectivity of traditional trend analysis based on linear trends. The sum of squares of the residuals between the estimated and actual values was calculated to obtain the turning point of the offset trend.

To assess global and regional disparities in age-standardized DALY rates for early-onset IHD, we calculated the Slope Inequality Index (SII) and the CI. The SII, an econometric measure, evaluates health disparities between the poorest subgroups by employing weighted regression models that factor in socioeconomic determinants like education and wealth. Conversely, the CI measures relative inequality by revealing the degree to which health indicators are concentrated among disadvantaged populations.

### Decomposition analysis

A method of robust decomposition analysis, which was detailed in prior studies,^22^, ^23^, ^24^ was employed. By taking into account 3 crucial elements, namely the demographic composition (which is characterized by a shift toward a greater proportion of older people, also known as population aging), population growth, and age-specific rates, the net effect of population aging was separated from population growth and change in age-specific rates. These terms were calculated with 1990 being the reference year.

All data were analyzed with R, version 4.2.2 (R Core Team). The Guidelines for Accurate and Transparent Health Estimates Reporting (GATHER) statement was adhered to when presenting the findings.^25^

### Risk factors

This study calculated the number of cases of DALY burden due to various IHD risk factors. We included 25 risk factors, grouped into 3 broad categories, namely behavioral, environmental, and metabolic factors, that contribute to the substantial DALY burden associated with early-onset IHD. These risk factors include behavioral patterns such as a diet low in fruits, vegetables, nuts, and seeds, whole grains, polyunsaturated fatty acids, fiber, legumes, and seafood omega-3 fatty acids, a diet high in red meat, processed meat, sodium, sugar-sweetened beverages and trans fatty acids, smoking, and low physical activity. In addition, environmental factors such as particulate matter pollution, low temperature, high temperature, secondhand smoke, and metabolic factors such as high body mass index, kidney dysfunction, high low-density lipoprotein cholesterol (LDL-C), high systolic blood pressure, and high fasting plasma glucose contribute to the development of IHD. We counted the number of cases in which these risk factors contributed to the burden of DALY between men and women and across SDI countries. Further information regarding the definitions and relative risks of these risk factors for IHD can be found elsewhere.^26^

## Results

### Incidence burden on early onset IHD between 1990 to 2019

The worldwide incidence of early-onset IHD reached 5.34 million (95% uncertainty interval [UI]: 3.96-6.96) cases in 2019. Additionally, the age-standardized incidence rate (ASIR) of early-onset IHD was recorded at 143.59 (95% UI: 106.28-187.54) in 2019. The EAPC reflects trends in ASIR of early-onset IHD. From 1990 to 2019, the EAPC for global incidence was declined (EAPC: −0.65%, 95% CI: −0.79 to −0.52) (Table 1).

**Table 1:** The Cases and ASRs of Prevalence and Incidence for Early-Onset Ischemic Heart Disease by SDI Region, Gender, and Region in 1990 and 2019, and EAPC in ASRs per 100,000, From 1990 to 2019

	Prevalence					Incidence				
	Cases, 1990 × 10^4^ (95% UI)	Cases, 2019 × 10^4^ (95% UI)	ASPR, 1990 per 100,000 (95% UI)	ASPR, 2019 per 100,000 (95% UI)	EAPC 1990-2019 (95% CI)	Cases, 1990 × 10^4^ (95% UI)	Cases, 2019 × 10^4^ (95% UI)	ASIR, 1990 per 100,000 (95% UI)	ASIR, 2019 per 100,000 (95% UI)	EAPC 1990-2019 (95% CI)
Global	2,485.67	4,754.65	1,263.67	1,265.85	−0.06	300.47	533.75	152.04	143.59	−0.65
	(2,085.51-2,984.70)	(4,004.52-5,693.72)	(1,061.43-1,516.01)	(1,065.91-1,515.88)	(−0.11 to −0.01)	(219.15-396.19)	(395.84-696.10)	(111.45-199.57)	(106.28-187.54)	(-0.79 to −0.52)
SDI region										
High SDI	445.24	558.75	1,101.08	959.29	−1.14	64.08	64.00	161.16	115.12	−2.95
	(379.59-523.95)	(486.68-643.59)	(938.87-1,295.42)	(834.37-1,106.15)	(-1.29 to −1.00)	(46.34-85.06)	(47.70-83.08)	(116.40-214.09)	(84.86-150.51)	(-3.18 to −2.72)
Low-middle SDI	427.65	948.78	1,273.27	1,342.57	0.36	57.77	126.96	169.40	179.34	0.34
	(359.53-512.72)	(797.90-1,136.25)	(1,071.75-1,525.31)	(1,129.65-1,606.93)	(0.27-0.45)	(42.40-76.04)	(94.68-164.78)	(125.62-221.20)	(134.38-231.94)	(0.11, 0.56)
High-middle SDI	700.12	1,155.33	1,362.41	1,322.44	−0.34	75.90	110.05	149.32	129.15	−1.33
	(581.51-852.28)	(957.84-1,407.56)	(1,132.81-1,656.44)	(1,095.56-1,611.26)	(-0.41 to −0.27)	(55.87-99.72)	(81.40-143.72)	(109.88-196.06)	(95.83-169.62)	(-1.49 to −1.16)
Middle SDI	754.46	1716.69	1,324.80	1,362.10	0.21	80.08	179.30	138.00	143.67	0.23
	625.49-919.31)	(1,434.67-2074.97)	(1,100.72-1,612.11)	(1,137.54-1,646.76)	(0.16-0.26)	(58.24-106.26)	(132.93-233.66)	(101.41-181.52)	(106.19-187.70)	(0.12, 0.34)
Low SDI	156.74	372.08	1,161.66	1,225.23	0.36	22.45	53.04	162.15	169.91	0.20
	(132.72-185.82)	(315.10-441.61)	(985.08-1,375.39)	(1,039.20-1,452.30)	(0.32-0.41)	(16.28-29.78)	(38.55-70.07)	(119.12-213.03)	(125.23-222.10)	(0.07, 0.37)
Sex										
Female	1,445.10	2,845.25	717.46	727.60	149.93	149.93	276.84	73.63	71.03	−0.30
	(1,210.31-1742.04)	(2,391.20-3,418.28)	(601.35-864.53)	(611.36-874.19)	(108.66-199.14)	(108.66-199.14)	(203.72-364.25)	(53.56-97.51)	(52.15-93.62)	(-0.36 to −0.23)
Male	1,040.57	1909.40	546.21	538.24	150.54	150.54	256.90	78.41	72.56	−0.35
	(875.21-1,242.66)	(1,613.32-2,275.45)	(460.08-651.48)	(454.56-641.69)	(110.49-197.05)	(110.49-197.05)	(192.12-331.85)	(57.88-102.06)	(54.13-93.92)	(-0.42 to −0.28)
Region										
East Asia	640.39	1,371.24	1,370.88	1,425.11	0.23	45.27	89.33	94.86	95.04	0.07
	(508.08-812.16)	(1,095.94-1,732.07)	(1,090.50-1,736.71)	(1,134.81-1,802.03)	(0.10-0.36)	(31.93-61.49)	(64.66-118.37)	(67.58-127.74)	(68.00-127.15)	(-0.01 to 0.14)
Southeast Asia	137.82	316.51	933.98	950.76	0.17	14.30	29.57	94.23	89.81	−0.52
	(113.84-168.79)	(261.75-387.35)	(772.90-1,143.08)	(785.95-1,163.71)	(0.10-0.23)	(10.06-19.51)	(21.21-39.55)	(67.18-127.08)	(64.37-120.16)	(-0.67 to −0.37)
Oceania	2.55	6.50	1,392.90	1,439.75	0.26	0.24	0.61	128.10	131.05	0.20
	(2.06-3.20)	(5.23-8.15)	(1,129.06-1,751.76)	(1,162.56-1,803.89)	(0.16-0.36)	(0.17-0.33)	(0.43-0.83)	(91.44-171.73)	(93.39-175.89)	(0.13-0.27)
Caribbean	22.84	42.08	1861.60	1832.81	−0.09	3.33	6.03	264.35	264.27	0.00
	(19.90-26.29)	(36.81-48.29)	(1,622.32-2,142.45)	(1,602.32-2,105.06)	(−0.11 to −0.06)	(2.41-4.40)	(4.40-7.92)	(192.48-347.25)	(192.50-347.36)	(-0.07 to 0.06)
Central Asia	44.10	75.05	1,772.43	1,726.82	−0.30	6.68	11.68	270.64	272.57	−0.26
	(37.97-51.47)	(65.12-87.26)	(1,527.81-2,066.06)	(1,499.47-2,007.00)	(−0.37 to −0.22)	(5.04-8.66)	(8.82-15.04)	(204.06-350.58)	(206.34-350.51)	(-0.50 to −0.01)
Eastern Europe	200.58	218.48	1,497.93	1,565.67	−0.02	27.81	29.28	218.76	226.22	−0.31
	(168.36-240.18)	(183.12-262.70)	(1,259.92-1,788.63)	(1,314.92-1,876.86)	(−0.15-0.10)	(20.90-35.84)	(21.96-37.80)	(162.54-284.16)	(168.64-293.68)	(-0.69 to 0.06)
Western Europe	221.75	241.80	1,099.91	942.13	−1.24	30.53	28.81	156.40	118.74	−2.03
	(188.17-261.35)	(209.97-279.97)	(934.30-1,294.68)	(817.72-1,090.62)	(−1.42 to −1.07)	(22.47-39.79)	(21.34-37.44)	(114.52-204.51)	(86.81-155.63)	(-2.25 to −1.81)
Central Europe	98.05	93.36	1,515.64	1,303.33	−1.23	9.66	8.83	154.62	128.65	−1.92
	(80.19-122.41)	(34.21-114.96)	(1,241.29-1,889.47)	(1,081.83-1,602.70)	(−1.32 to −1.14)	(7.08-12.67)	(6.66-11.40)	(112.87-203.43)	(95.90-167.42)	(-2.16 to −1.69)
Australasia	13.61	20.37	1,479.98	1,324.69	−0.96	2.27	3.20	247.71	214.53	−1.26
	(11.86-15.55)	(17.82-23.15)	(1,289.47-1,688.82)	(1,157.15-1,506.48)	(−1.09 to −0.83)	(1.64-2.99)	(2.35-4.20)	(178.92-326.93)	(157.08-282.64)	(-1.45 to −1.06)
High-income Asia Pacific	59.95	60.78	651.25	548.45	−1.36	7.35	7.17	82.13	68.14	−1.71
	(50.87-70.97)	(52.11-71.08)	(552.40-771.03)	(468.92-642.81)	(−1.50 to −1.23)	(5.21-9.89)	(5.14-9.59)	(58.04-110.73)	(48.10-92.16)	(-1.89 to −1.53)
Southern Latin America	11.35	16.27	558.01	500.81	−0.77	1.61	2.13	80.16	67.34	−1.59
	(9.72-13.29)	(14.03-18.96)	(478.26-652.66)	(431.49-583.32)	(−0.82 to −0.72)	(1.14-2.17)	(1.50-2.90)	(56.97-108.10)	(47.23-91.67)	(-1.71 to −1.47)
High-income North America	159.99	186.71	1,244.96	920.96	−2.48	26.49	20.10	207.88	105.35	−5.81
	(134.79-190.21)	(160.28-217.39)	(1,049.45-1,479.76)	(788.46-1,075.58)	(−2.69 to −2.28)	(18.45-36.16)	(15.35-25.69)	(145.00-283.47)	(79.28-135.84)	(-6.34 to −5.28)
Andean Latin America	5.99	14.67	539.31	547.51	0.12	0.44	1.01	37.76	37.60	−0.08
	(4.73-7.69)	(11,62-18.93)	(426.76-694.40)	(434.05-706.73)	(0.01-0.22)	(0.28-0.65)	(0.65-1.45)	(24.99-53.80)	(25.06-53.55)	(-0.19 to 0.04)
Central Latin America	48.92	112.67	1,045.37	978.95	−0.44	5.98	12.81	123.17	112.29	−0.77
	(41.20-58.54)	(95.03-134.77)	(882.07-1,249.28)	(825.72-1,170.82)	(−0.54 to −0.34)	(4.22-8.09)	(9.15-17.19)	(88.22-164.56)	(80.27-150.42)	(-0.84 to −0.71)
North Africa and Middle East	216.69	559.80	2,289.49	2,251.13	−0.07	33.20	83.36	348.40	333.67	−0.50
	(189.43-248.47)	(489.62-640.40)	(2,005.45-2,620.24)	(1,971.50-2,571.52)	(−0.14 to 0.00)	(25.13-42.62)	(63.34-106.90)	(266.12-444.25)	(254.80-426.52)	(-0.64 to −0.36)
Tropical Latin America	44.31	96.83	882.08	856.17	−0.26	3.95	8.11	75.37	71.72	−0.25
	(35.42-56.40)	(77.76-122.41)	(705.59-1,124.11)	(686.56-1,083.72)	(−0.33 to −0.19)	(2.75-5.40)	(5.89-10.76)	(53.51-101.77)	(51.90-95.46)	(-0.30 to −0.19)
Eastern sub-Saharan Africa	42.11	106.26	963.38	1,031.99	0.47	5.49	13.54	118.62	123.68	0.26
	(35.05-51.20)	(88.37-129.44)	(802.90-1,171.07)	(859.43-1,256.65)	(0.37-0.57)	(3.85-7.46)	(9.48-18.38)	(84.71-158.90)	(88.13-165.55)	(0.18-0.34)
Western sub-Saharan Africa	43.86	119.46	902.54	997.07	0.77	5.90	15.80	117.00	125.89	0.55
	(37.11-52.19)	(100.71-143.75)	(765.60-1,071.59)	(841.89-1,190.37)	(0.66-0.88)	(4.15-7.99)	(11.11-21.35)	(83.49-156.56)	(89.96-168.00)	(0.46-0.63)
Southern sub-Saharan Africa	17.21	34.41	1,146.04	1,097.94	−0.50	2.57	4.97	162.82	154.75	−0.41
	(14.39-20.60)	(28.86-41.01)	(958.86-1,370.87)	(920.69-1,309.35)	(−0.72 to −0.28)	(1.82-3.45)	(3.55-6.63)	(116.86-216.47)	(111.19-205.55)	(-0.54 to −0.27)
South Asia	440.73	1,029.52	1,338.55	1,426.51	0.37	65.32	152.32	195.16	211.05	0.46
	(369.26-528.31)	(864.62-1,234.21)	(1,122.22-1,604.03)	(1,198.76-1709.19)	(0.20-0.54)	(48.05-85.55)	(113.91-197.57)	(144.94-253.81)	(158.61-272.77)	(0.18-0.75)
Central sub-Saharan Africa	12.88	31.89	921.47	901.95	−0.22	2.07	5.07	141.47	137.36	−0.77
	(11.05-15.04)	(27.42-37.23)	(791.67-1,074.97)	(777.72-1,050.54)	(−0.26 to −0.17)	(1.46-2.78)	(3.58-6.84)	(101.43-188.09)	(98.59-183.33)	(-0.84 to −0.71)

ASR = age-standardized rate; ASIR = age-standardized incidence rate; ASPR = age-standardized prevalence rate; EAPC = estimated annual percentage change; SDI = sociodemographic index; UI = uncertainty interval.

#### Sociodemographic indices level

In 2019, the middle SDI recorded the highest numbers of incidence (1.79 million, 95% UI: 1.33-2.34) and the low-middle SDI had the highest incidence rate (ASIR: 179.34 per 100,000, 95% UI: 134.28-231.94). From 1990 to 2019, positive trends in the ASIR were observed in countries with middle SDI (EAPC: 0.23%, 95% CI: 0.12-0.34), countries with low-middle SDI (EAPC: 0.34%, 95% CI: 0.11-0.56), and countries with low SDI (EAPC: 0.20%, 95% CI: 0.07-0.37). There was a decrease in the ASIR of early-onset IHD in countries with high SDI (EAPC: −2.95%, 95% CI: −3.18 to −2.72) and high-middle (EAPC: −1.33%, 95% CI: −1.49 to −1.16) (Table 1).

#### Regional level

In 2019, South Asia showed the highest number of incidence (1.52 million, 95% UI: 1.14-1.98) and North Africa and Middle East showed the highest incidence rate (ASIR: 333.67 per 100,000, 95% UI: 254.80-426.52). From 1990 to 2019, the incidence rate increased in a small number of areas. The largest increases occurred in Western sub-Saharan Africa (EAPC: 0.55%, 95% CI: 0.46-0.63), South Asia (EAPC: 0.46%, 95% CI: 0.18-0.75), and Eastern sub-Saharan Africa (EAPC: 0.26%, 95% CI: 0.18-0.34) (Table 1).

Conversely, East Asia displayed the smallest increase (EAPC: 0.07%, 95% CI: −0.01-0.14). In terms of ASIR, the largest decrease was observed in high-income North America (EAPC: −5.81%, 95% CI: −6.34 to −5.28). On the other hand, the EAPC of Caribbean was 0.00 (95% CI: −0.07-0.06), implying a relatively stable rate of early-onset IHD incidence in that region (Table 1).

#### Sex pattern

In 2019, the number of incidence cases in women was greater than the number in men, which takes into account the possibility that the age definition of early-onset IHD is greater in women than in men. Specifically, men had 2.57 million (95% UI: 1.92-3.32) incidence cases. Women had 2.77 million (95% UI: 2.04-3.64) for incidence cases. The ASIR for 2019 is higher for males than females (72.56 vs 71.03). The burden of incidence for both was gradually decreasing in both men and women (men: EAPC: −0.35%, women: EAPC: −0.30%) (Table 1).

**Figure 1:**
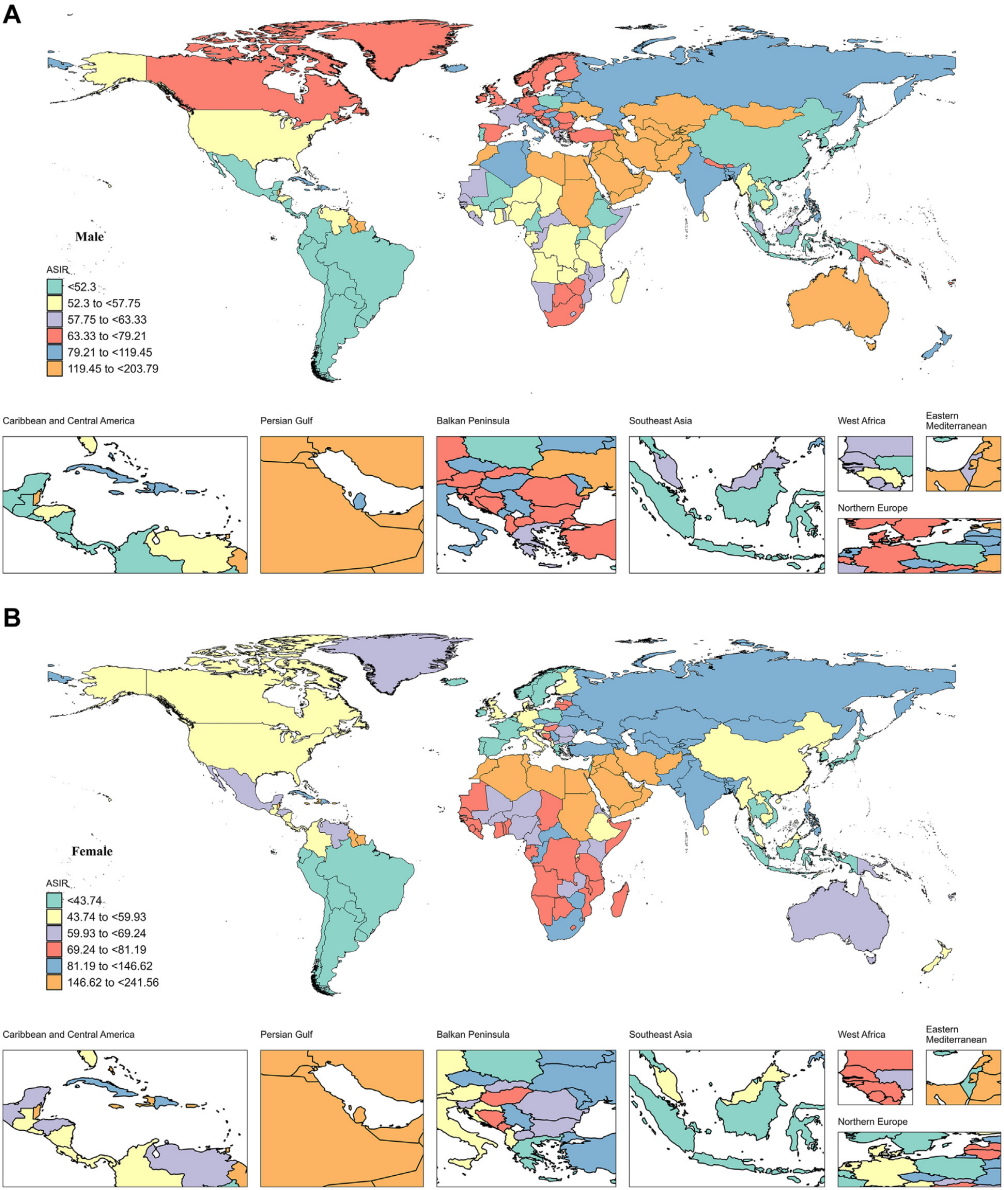
Global ASIR of Early-Onset IHD by Sex, 2019 The age-standardized incidence rate of males (A) and females (B) early-onset ischemic heart disease in 204 countries and territories in 2019. ASIR = age-standardized incidence rate.

In 2019, the highest ASIR (119.45 to < 203.79) in males was concentrated in Australia, Southwest Asia, and parts of northeast Africa. For females, the highest ASIR (146.62 to <241.56) was concentrated in parts of Southwest Asia, Northeast Africa, and northern Africa. The lowest ASIR (<52.3), males were concentrated in South-East, Central Asia, and most of South America. In terms of the lowest ASIR values (<43.74), females were concentrated in Southeast Asia, small parts of South America, and parts of Europe (Figures 1A and 1B).

**Figure 2:**
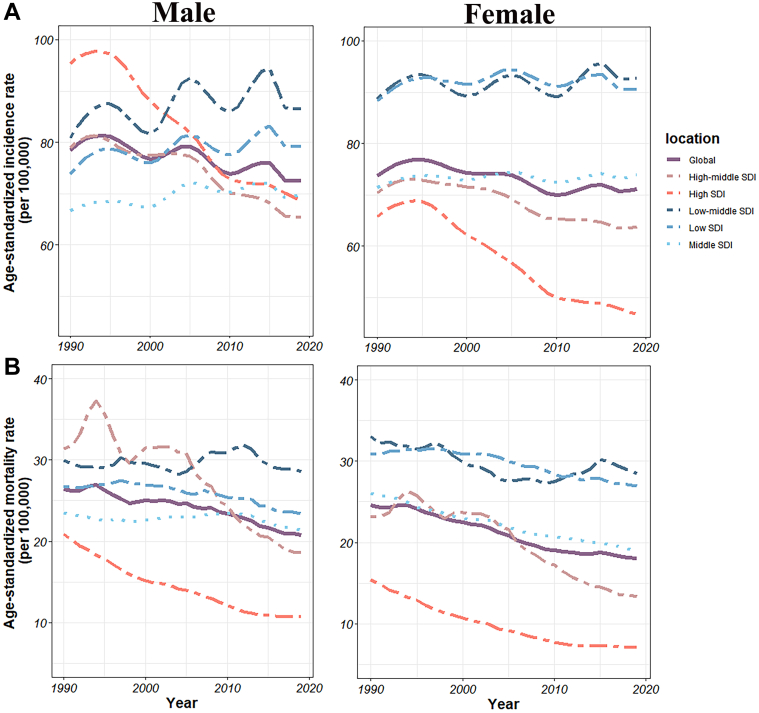
Trends in ASIR and ASMR of Early-Onset IHD, 1990 to 2019 Temporal trend of age-standardized incidence rate (A) and age-standardized mortality rate (B) (per 100,000 persons) for the burden of early-onset ischemic heart disease, globally and among different sociodemographic index quintiles from 1990 to 2019. SDI = sociodemographic index.

Globally, ASIR has shown an overall decreasing trend from 1990 to 2019 for both men and women, with a greater decrease for men than for women. Among males, ASIR decreased the most in high SDI, while ASIR in other SDI countries showed an increase followed by a decrease. In addition, high-middle SDI showed an overall decreasing trend, while middle, low-middle, and low SDI showed an overall increasing trend. Among females, high SDI showed the greatest decrease in ASIR, high-middle SDI showed an overall decreasing trend, while middle, low-middle, and low SDI showed an overall increasing trend. In addition, among females, ASIR was much greater in low and low-mid SDI countries than in other SDI countries (Figure 2).

**Figure 3:**
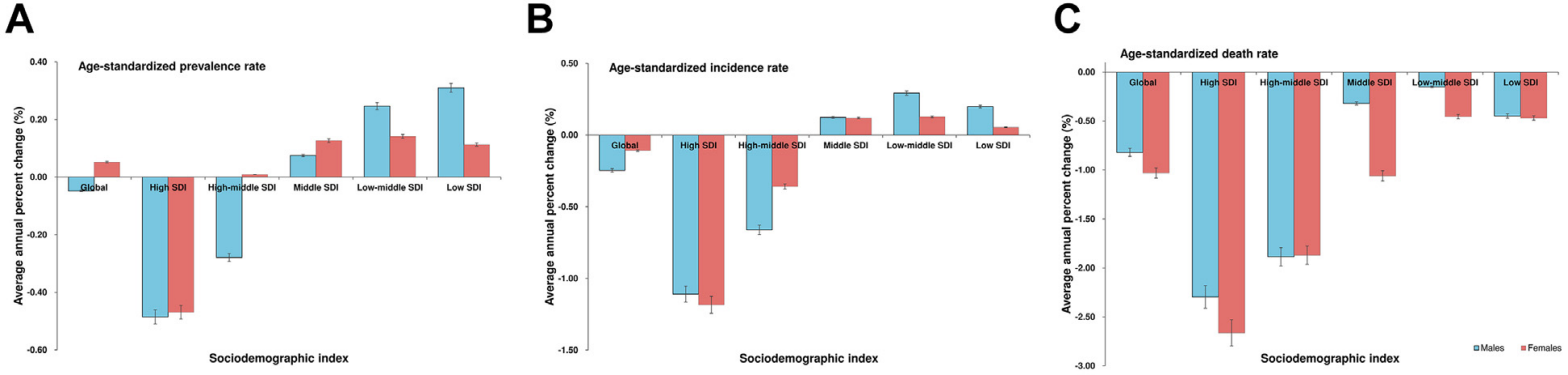
AAPC of ASR Metrics for Early-Onset IHD, 1990 to 2019 Average annual percent change of age-standardized prevalence rate (A), age-standardized incidence rate (B), and age-standardized death rate (C) for the burden of early onset ischemic heart disease, global and countries with five SDI (high, high-middle, middle, low-middle, and low) from 1990 to 2019. Black bars represent 95% confidence intervals. Abbreviation as in Figure 2.

When looking at specific SDI areas, the average annual percent change of ASIR declined globally in both males and females and also declined in high and high-middle SDI countries, while rising in low, low-middle, and middle SDI countries (Figure 3B).

### Prevalence, DALY, and death of early onset IHD, 1990 to 2019

In 2019, the global prevalence cases of early-onset IHD reached 47.55 million (95% UI: 40.05-56.94), with an age-standardized prevalence rate (ASPR) of 1,265.85 (95% UI: 1,065.91-1,515.88) and EAPC of −0.06% (95% CI: −0.11 to −0.01) from 1990 to 2019. The number of DALY cases of early-onset IHD reached 58.48 million globally (95% UI: 52.65-64,67), with an age-standardized DALY rate of 1,597.04 (95% UI: 1,437.98-1767.43) and EAPC of −1.97% (95% CI: −2. 13 to −1.80) from 1990 to 2019. The worldwide death of early-onset IHD reached 1.44 million (95% UI: 1.29-1.59) cases in 2019. The age-standardized death rate (ASDR) of early-onset IHD was recorded at 38.80 per 100,000 (95% UI: 34.87-42.89) in 2019. From 1990 to 2019, the EAPC for global death was negative (−2.08%, 95% CI: −2.25 to −1.91) (Tables 1 and 2).

**Table 2:** The Cases and ASRs of DALY and Deaths for Early-Onset Ischemic Heart Disease by SDI Region, Gender, and Region in 1990 and 2019, and EAPC in ASRs per 100,000, From 1990 to 2019

	DALY					Deaths				
	Cases, 1990, ×10^4^ (95% UI)	Cases, 2019, ×10^4^ (95% UI)	ASRs, 1990 per 100,000 (95% UI)	ASRs, 2019 per 100,000 (95% UI)	EAPC, 1990-2019 (95% CI)	Cases, 1990, ×10^4^ (95% UI)	Cases, 2019, ×10^4^ (95% UI)	ASDR, 1990 per 100,000 (95% UI)	ASDR, 2019 per 100,000 (95% UI)	EAPC 1990-2019 (95% CI)
Global	4,091.56	5,848.16	2,054.27	1,597.04	−1.97	100.37	143.76	51.00	38.80	−2.08
	(3,819.75-4,380.63)	(5,265.18-6,468.66)	(1,921.06-2,195.54)	(1,437.98-1,767.43)	(−2.13 to −1.80)	(93.98-107.30)	(129.19-158.84)	(47.81-54.45)	(34.87-42.89)	(−2.25 to −1.91)
SDI region										
High SDI	568.51	411.40	1,429.45	726.49	−4.88	14.61	10.40	36.23	17.73	−5.19
	(551.76-586.27)	(383.97-446.58)	(1,386.79-1,474.53)	(675.80-792.33)	(−5.15 to −4.60)	(14.23-15.02)	(9.73-11.25)	(35.28-37.24)	(16.53-19.25)	(−5.48 to −4.89)
Low-middle SDI	(900.43)	1,661.44	2,535.26	2,309.67	−0.47	21.64	40.57	62.97	57.13	−0.43
	(784.22-1,034.57)	(1,410.24-1939.96)	(2,213.55-2,903.79)	(1,962.31-2,693.93)	(−0.77 to −0.18)	(18.86-24.81)	(34.38-47.29)	(54.97-72.03)	(48.45-66.53)	(−0.74 to −0.12)
High-middle SDI	1,098.30	1,089.72	2,195.63	1,320.55	−4.48	27.56	27.16	54.58	31.93	−4.65
	(1,021.81-1,167.66)	(982.65-1,206.21)	(2042.83-2,333.10)	(1,189.37-1,463.84)	(−5.10 to −3.86)	(25.78-29.18)	(24.45-30.04)	(51.01-57.78)	(28.72-35.36)	(−5.30 to −4.01)
Middle SDI	1,202.76	2043.59	2010.95	1,672.49	−1.16	28.65	50.08	49.51	40.45	−1.18
	(1,098.69-1,315.45)	(1,812.77-2,286.17)	(1,840.89-2,195.37)	(1,483.71-1,871.59)	(−1.28 to −1.04)	(26.16-31.32)	(44.29-56.07)	(45.38-54.05)	(35.78-45.30)	(−1.31 to −1.05)
Low SDI	319.20	638.09	2,267.65	1,991.15	−1.04	7.84	15.45	57.67	50.31	−1.08
	(263.93-383.30)	(533.32-756.27)	(1,879.34-2,711.43)	(1,670.11-2,351.39)	(−1.22 to −0.86)	(6.48-9.40)	(12.93-18.30)	(47.68-68.96)	(42.23-59.41)	(−1.24 to −0.91)
Sex										
Female	1831.82	2,582.16	894.11	665.57	−1.24	49.75	70.41	24.60	18.01	−1.24
	(1,691,89.96-1,985.23)	(2,307.63-2,849.93)	(827.01-967.36)	(594.60-734.91)	(−1.32 to −1.16)	(46.20-53.71)	(62.91-77.67)	(22.87-26.54)	(16.09-19.88)	(-1.32 to −1.16)
Male	2,259.73	3,265.99	1,160.16	931.47	−0.79	50.62	73.35	26.60	20.79	−0.85
	(2,127.86-2,395.40)	(2,957.55-3,618.72)	(1,094.05-1,228.19)	(843.38-1,032.53)	(−0.87 to −0.70)	(47.78-53.59)	(66.27-81.16)	(24.94-27.91)	(18.78-23.01)	(−0.93 to −0.76)
Region										
East Asia	629.85	924.06	1,288.42	1,032.77	−1.25	14.57	22.00	30.63	23.75	−1.19
	(506.81-758.65)	(734.70-1,142.08)	(1,038.67-1,550.31)	(822.18-1,273.65)	(−1.51 to −0.98)	(11.67-17.62)	(17.25-27.62)	(24.56-37.02)	(18.66-29.74)	(−1.51 to −0.86)
Southeast Asia	292.13	562.67	1901.13	1713.04	−0.61	6.98	13.62	46.86	41.26	−0.68
	(249.52-342.75)	(468.37-668.78)	(1,630.53-2,218.12)	(1,425.31-2,035.36)	(−0.82 to −0.41)	(5.96-8.19)	(11.34-16.24)	(40.12-54.71)	(34.33-49.12)	(−0.90 to −0.47)
Oceania	7.58	19.50	3,975.19	4,155.09	0.60	0.18	0.47	98.28	103.09	0.58
	(5.51-10.51)	(13.74-27.54)	(2,898.96-5,476.99)	(2,938.22-5,844.27)	(0.43-0.77)	(0.13-0.25)	(0.33-0.66)	(71.19-135.51)	(72.71-144.87)	(0.41-0.74)
Caribbean	29.59	39.61	2,401.52	1757.26	−2.16	0.75	1.01	61.57	44.07	−2.27
	(26.28-33.60)	(30.54-50.34)	(2,140.32-2,715.86)	(1,348.97-2,236.04)	(−2.68 to −1.65)	(0.66-0.84)	(0.78-1.27)	(54.95-69.53)	(34.03-55.81)	(−2.78 to −0.23)
Central Asia	87.83	136.27	3,667.23	3,200.17	−2.34	2.26	3.53	93.04	82.06	−2.32
	(83.60-91.96)	(119.02-156.03)	(3,492.02-3,837.69)	(2,796.65-3,663.00)	(−3.27 to −1.41)	(2.15-2.36)	(3.08-4.03)	(88.74-97.21)	(71.71-93.88)	(−3.26 to −1.39)
Eastern Europe	387.10	336.80	3,267.65	2,806.80	−2.92	10.22	8.85	82.18	69.97	−2.98
	(350.49-404.93)	(285.11-398.19)	(2,928.20-3,428.88)	(2,368.20-3,333.39)	(−4.41 to −1.42)	(9.33-10.65)	(7.50-10.44)	(74.18-85.97)	(59.10-82.90)	(−4.48 to −1.47)
Western Europe	251.96	115.71	1,283.15	466.18	−7.56	6.51	2.91	32.23	11.30	−7.83
	(243.98-260.38)	(109.80-188.02)	(1,242.35-1,326.37)	(442.36-494.04)	(−7.87 to −7.25)	(6.32-6.71)	(2.77-3.08)	(31.28-33.23)	(10.74-11.96)	(−8.16 to −7.50)
Central Europe	175.84	79.03	2,912.45	1,175.34	−7.33	4.51	2.04	72.59	29.28	−7.39
	(170.25-181.580	(66.88-92.13)	(2,820.65-3,006.70)	(993.38-1,371.45)	(−7.69 to −6.97)	(4.37-4.64)	(1.72-2.39)	(70.40-74.82)	(24.66-34.30)	(−7.76 to −7.02)
Australasia	12.68	7.04	1,379.35	470.33	−8.04	0.33	0.17	35.58	11.34	−8.51
	(11.91-13.46)	(6.28-7.83)	(1,296.79-1,463.33)	(419.26-523.65)	(−8.53 to −7.55)	(0.31-0.35)	(0.16-0.19)	(33.52-37.70)	(10.10-12.60)	(−9.06 to −7.96)
High-income Asia Pacific	73.62	33.08	824.22	310.73	−6.78	1.80	0.80	19.70	7.26	−6.89
	(66.66-79.64)	(30.47-36.21)	(742.14-894.27)	(285.09-340.70)	(−7.23 to −6.32)	(1.65-1.93)	(0.74-0.88)	(17.99-21.22)	(6.67-7.96)	(−7.33 to −6.44)
Southern Latin America	30.60	23.21	1,527.03	724.01	−5.18	0.78	0.59	38.38	18.15	−5.18
	(28.00-33.38)	(20.57-25.92)	(1,398.77-1,664.50)	(642.33-808.07)	(-5.50 to −4.86)	(0.71-0.84)	(0.53-0.66)	(35.26-41.75)	(16.13-20.21)	(−5.48 to −4.89)
High-income North America	222.31	190.82	1,767.92	963.06	−4.42	5.78	5.02	45.66	24.20	−4.71
	(215.79-229.80)	(180.59-204.30)	(1,717.10-1,827.25)	(909.60-1,033.18)	(−4.67 to −4.16)	(5.63-5.96)	(4.76-5.37)	(44.45-47.08)	(22.91-25.93)	(−4.99 to −4.43)
Andean Latin America	15.74	19.73	1,316.34	729.95	−4.18	0.37	0.47	32.09	17.35	−4.29
	(12.68-19.39)	(14.69-26.09)	(1,065.92-1,614.19)	(544.29-964.31)	(−4.64 to −3.72)	(0.30-0.45)	(0.35-0.62)	(26.03-39.31)	(12.88-22.94)	(−4.76 to −3.82)
Central Latin America	74.75	125.96	1,532.89	1,124.00	−2.49	1.80	3.10	38.48	27.49	−2.61
	(71.54-78.37)	(104.98-150.65)	(1,470.81-1,604.49)	(936.58-1,344.32)	(−2.91 to −2.08)	(1.73-1.88)	(2.57-3.71)	(37.00-40.25)	(22.81-32.98)	(−3.00 to −2.21)
North Africa and Middle East	453.78	708.07	4,543.08	2,754.59	−3.91	11.16	17.55	115.82	70.20	−3.93
	(402.17-516.79)	(581.34-866.60)	(4,044.85-5,152.07)	(2,266.81-3,359.71)	(−4.09 to −3.73)	(9.92-12.65)	(14.4-21.41)	(103.31-130.82)-	(57.89-85.32)	(−4.11 to −3.75)
Tropical Latin America	117.41	132.28	2,306.07	1,179.42	−4.52	2.90	3.32	58.13	29.25	−4.64
	(111.27-123.85)	(123.04-142.21)	(2,188.34-2,429.93)	(1,097.35-1,267.59)	(−4.67 to −4.38)	(2.75-3.05)	(3.09-3.57)	(55.25-61.25)	(27.18-31.39)	(−4.78 to −4.51)
Eastern sub-Saharan Africa	62.48	119.76	1,359.57	1,093.47	−1.88	1.49	2.81	33.89	27.14	−1.90
	(48.63-81.10)	(91.22-152.84)	(1,061.98-1,751.62)	(835.68-1,390.23)	(−2.06 to −1.70)	(1.15-1.94)	(2.13-3.59)	(26.26-43.82)	(20.63-34.61)	(−2.07 to −1.73)
Western sub-Saharan Africa	65.69	144.82	1,334.17	1,188.93	−0.97	1.65	3.62	34.82	30.95	−0.98
	(49.13-88.55)	(109.09-188.02)	(999.59-1,794.71)	(898.78-1,537.10)	(−1.26 to −0.67)	(1.23-2.22)	(2.72-4.69)	(26.01-46.89)	(23.34-40.04)	(−1.26 to −0.69)
Southern Sub-Saharan Africa	22.10	35.11	1,437.68	1,126.76	−1.43	0.53	0.89	35.48	28.74	−1.16
	(18.45-26.16)	(27.81-43.64)	(1,205.06-1,694.76)	(897.37-1,393.51)	(−2.33 to −0.52)	(0.44-0.63)	(0.71-1.10)	(29.76-41.83)	(22.93-35.42)	(−2.06 to −0.26)
South Asia	1,053.47	2042.39	2,966.72	2,749.21	−0.33	25.19	49.66	73.63	67.94	−0.31
	(901.23-1,218.46)	(1,668.22-2,457.00)	(2,541.92-3,424.67)	(2,244.97-3,306.02)	(−0.71 to 0.04)	(21.54-29.11)	(40.38-59.80)	(62.97-85.02)	(55.23-81.79)	(-0.69 to 0.08)
Central sub-Saharan Africa	25.06	52.23	1793.99	1,461.23	−1.58	0.64	1.32	46.74	38.32	−1.60
	(16.87-36.51)	(33.48-78.48)	(1,218.32-2,591.86)	(940.07-2,186.73)	(−1.80 to −1.36)	(0.43-0.93)	(0.84-1.98q)	(31.63-67.67)	(24.49-57.50)	(−1.81 to −1.39)

ASDR = age-standardized deaths rate; DALY = disability-adjusted life year; other abbreviations as in Table 1.

#### Sociodemographic indices level

In 2019, the middle SDI recorded the highest number of prevalence cases (17.17 million, 95% UI: 14.35-20.75), the highest number of DALY (20.44 million, 95% UI: 18.13-22.86), and the highest number of death cases (0.50 million, 95% UI: 0.44-0.56). And the middle SDI had the highest ASPR (ASPR = 1,362.10 per 100,000, 95% UI: 1,137.54-1,646.76). The low-middle SDI had the highest age-standardized DALY rate (2,309.67 per 100,000, 95% UI: 1962.31-2,693.93) and the highest ASDR (57.13 per 100,000, 95% UI: 48.45-66.53) (Tables 1 and 2).

#### Regional level

In 2019, East Asia showed the highest number of prevalence cases (13.71 million, 95% UI: 10.96-17.32) and South Asia showed the highest number of DALY (20.42 million, 95% UI: 16.68-24.57), the highest number of death (0.50 million, 95% UI: 0.40-0.60). North Africa and Middle East had the highest prevalence rate (ASPR: 2,251.13, 95% UI: 1971.50-2,571.52), Oceania had the highest DALY rate (4,155.00, 95% UI: 2,938.22-5,844.27) and the highest death rate (ASDR: 103.09, 95% UI: 72.71-144.87). Prevalence of early-onset IHD decreased in most regions, with the highest increase in Western sub-Saharan Africa (EAPC: 0.77%, 95% CI: 0.66-0.88), Eastern sub-Saharan Africa (EAPC: 0.47%, 95% CI: 0.37-0.57), and South Asia (EAPC: 0.37%, 95% CI: 0.20-0.54) (Tables 1 and 2).

Conversely, the largest decrease in ASPR of IHD was observed in high-income North America (EAPC: −2.48%, 95% CI: −2.69 to −2.28), while the smallest decrease occurred in Eastern Europe (EAPC: −0.02%, 95% CI: −0.15-0.10) (Table 1).

Early-onset IHD DALY rates declined in most regions except Oceania, with the largest decreases in DALY rates in Australasia (EAPC: −8.04%, 95% CI: −8.53 to −7.55), Western Europe (EAPC: −7.56%, 95% CI: −7.87 to −7.25), and Central Europe (EAPC: −7.33%, 95% CI: −7.69 to −6.97). The EAPC for age-standardized DALY rates in region Oceania was 0.60% (95% CI: 0.43-0.77) (Table 2).

Early-onset IHD death rates declined in most regions except Oceania, with the largest decreases in Australasia (EAPC: −8.51%, 95% CI: −9.06 to −7.96), Western Europe (EAPC: −7.83%, 95% CI: −8.16 to −7.50), and Central Europe (EAPC: −7.39%, 95% CI: −7.76 to −7.02). The EAPC for ASDR in region Oceania was 0.58% (95% CI: 0.41-0.74) (Table 2).

#### Sex pattern

In 2019, the global number of prevalent cases was higher in women than in men (28.45 million vs 19.09 million). Males had a higher global number of DALY and death from early-onset IHD than females, with 32.66 million (95% UI: 29.58-36.19) DALYs and 0.73 million (95% UI: 0.66-0.81) deaths. For women, there were 25.82 million DALY (95% UI: 23.08-28.50) and 0.70 million (95% UI: 0.63-0.78) deaths (Tables 1 and 2).

In 2019, the highest ASPR in males (799.97 to <1,021.51) was concentrated in Australia, Southwest Asia, and parts of Northeast Africa. The highest ASPR for females (1,045.54 to <1,491.67) is concentrated in Southwest Asia and parts of Northern Africa. Males with the lowest ASPR (<396.62) were concentrated in Central Africa and small parts of South America. Females with the lowest ASPR (<413.27) were concentrated in Southeast Asia and most of South America (Central Illustration).

**Central Illustration:**
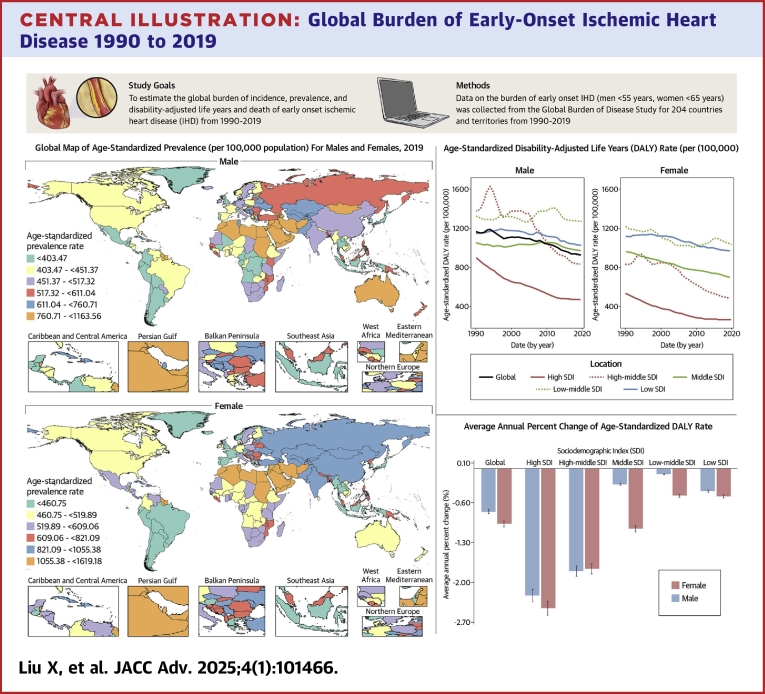
**Global Burden of Early-Onset Ischemic Heart Disease 1990 to 2019** (Left panel) World map of the age-standardized prevalence of early-onset ischemic heart disease globally in 2019, stratified by sex. (Upper left panel) Age-standardized disability-adjusted life year rate for early-onset ischemic heart disease globally as well as for the five sociodemographic index in 2019. (Upper right panel) Average annual percentage change in the age-standardized disability-adjusted life year rate for early-onset ischemic heart disease, globally and five sociodemographic index regions. IHD = ischemic heart disease; other abbreviations as in Figures 2 and 4.

Globally, from 1990 to 2019, ASPR has shown a decreasing trend in males and an increasing trend in females. The overall ASPR for females is higher than for males. Among males, ASPR declined the most in high SDI countries, with an overall downward trend in high-middle SDI countries and an upward trend in other SDI countries. Among females, ASPR decreased in high SDI countries, while trending up overall in high-middle, middle, low-middle, and low SDI countries (Supplemental Figure 1). Age-standardized DALY rate and age-standardized mortality rate showed a decreasing trend for both sexes, both globally and in any SDI countries. Moreover, the overall age-standardized DALY rate and age-standardized mortality rate were higher in men than in women (Central Illustration, Figure 2).

Globally, the average annual percent change for ASPR of SDI was positive for females and negative for males, from 1990 to 2019. The average annual percent change of ASPR in high-middle SDI was positive for women and negative for men. The average annual percent change of ASPR for both sexes in low-middle, low, and middle SDI was positive. And both sexes were negative for high SDI (Figure 3A). From 1990 to 2019, the average annual percent change of both the age-standardized DALY rate and ASDR were negative in all SDI countries (Central Illustration, Figure 3C).

We found that the standardized prevalence for females decreased significantly from 2005 to 2010 (average percent change [APC]: 0.14%) and the ASIR for males decreased significantly from 2014 to 2017 (APC: −1.57%). The ASPR for men decreased slightly from 2017 to 2019 (APC: −0.05%), whereas the ASPR for men increased slightly from 2014 to 2019 (APC: 0.04%). From 2002 to 2019, age-standardized DALY rate decreased for both males (2002-2011 APC: −0.81%, 2011-2016 APC: −1.75%, 2016-2019 APC: −0.57%) and females (2002-2009 APC: −1.87%, 2009-2019 APC: −0.56%). While ASDR decreased from 2003 to 2019 for males (2002-2011 APC: −0.88%, 2011-2017 APC: −1.71%, 2017-2019 APC: −0.31%) and females (2003-2009 APC: −2.04%, 2009-2019 APC: −0.55%) (Supplemental Figure 2).

DALY rates and death rates for both males and females show an overall downward trend from 1990 to 2019. DALY rates and death rates are highest for males at ages 45 to 54 and for females at ages 55 to 64 (Supplemental Figure 3).

Globally, significant absolute and relative SDI-associated inequalities in age-standardized DALY rate for IHD were observed in both men and women, with countries with lower SDI bearing a disproportionately higher burden (Supplemental Figure 4). From 1990 to 2019, the SII for age-standardized DALY rate for IHD in women showed worsening inequality in low SDI countries, increasing from −113.90 (95% CI: −618.84 to 391.05) to −416.52 (95% CI: −873.59 to 40.55) in women (Supplemental Figure 4B). Similarly, the concentration index for age-standardized DALY rates decreased from 0.33 (95% CI: 0.32-0.35) to 0.31 (95% CI: 0.30-0.32) in men and women. The concentration index for age-standardized DALY rates decreased from 0.32 (95% CI: 0.31-0.33) to 0.30 (95% CI: 0.29-0.30) (Supplemental Figure 4).

### Attributable risk factors for early onset IHD

**Figure 4:**
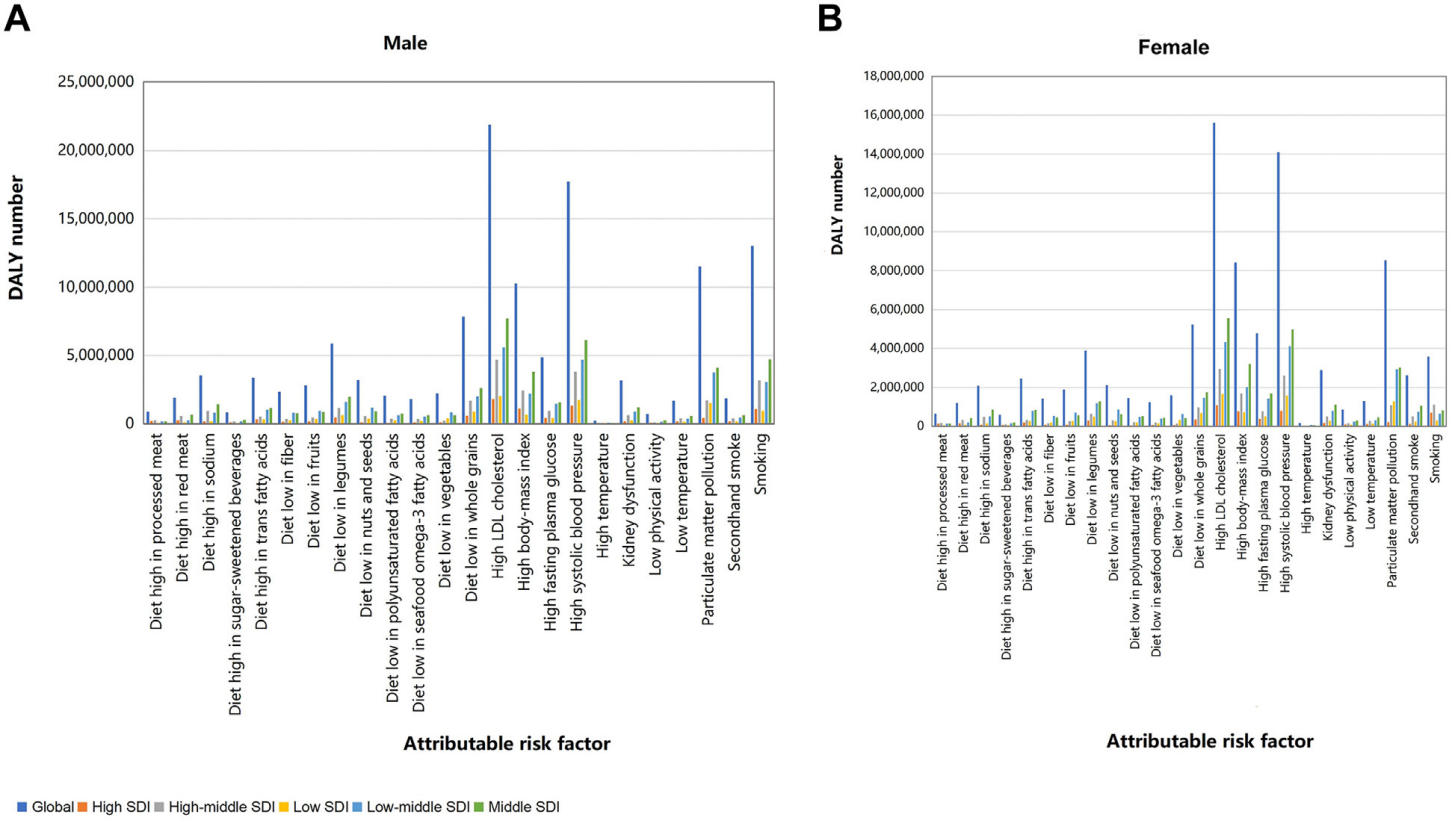
DALY Cases by Risk Factors and SDI Quintiles, 2019 The cases of disability-adjusted life year of males (A) and females (B) for early-onset ischemic heart disease attributable to 24 risk factors by global and different SDI quintiles in 2019. DALY = disability-adjusted life year; LDL = low-density lipoprotein; other abbreviation as in Figure 2.

In 2019, among the 24 risk factors associated with early-onset IHD, among men, the factor that globally contributes most to DALY was high LDL-C (21,876,994), followed by high systolic blood pressure (17,710,733) and smoking (13,035,504). Of these 3 risk factors, the largest number of people were affected in countries with high-middle SDI (6,250,008), low-middle SDI (8,579,934), and middle SDI (10,944,915) (Figure 4A). Among women, the factor with the greatest impact on global DALY was high LDL-C (15,605,502), followed by high systolic blood pressure (14,095,648) and particulate matter pollution (8,532,541). The burden of attributable risk was highest in high-middle SDI (4,362,795), low-middle SDI (7,201,599), and middle SDI countries (8,633,027) (Figure 4B).

### Decomposition analysis of early-onset IHD

**Figure 5:**
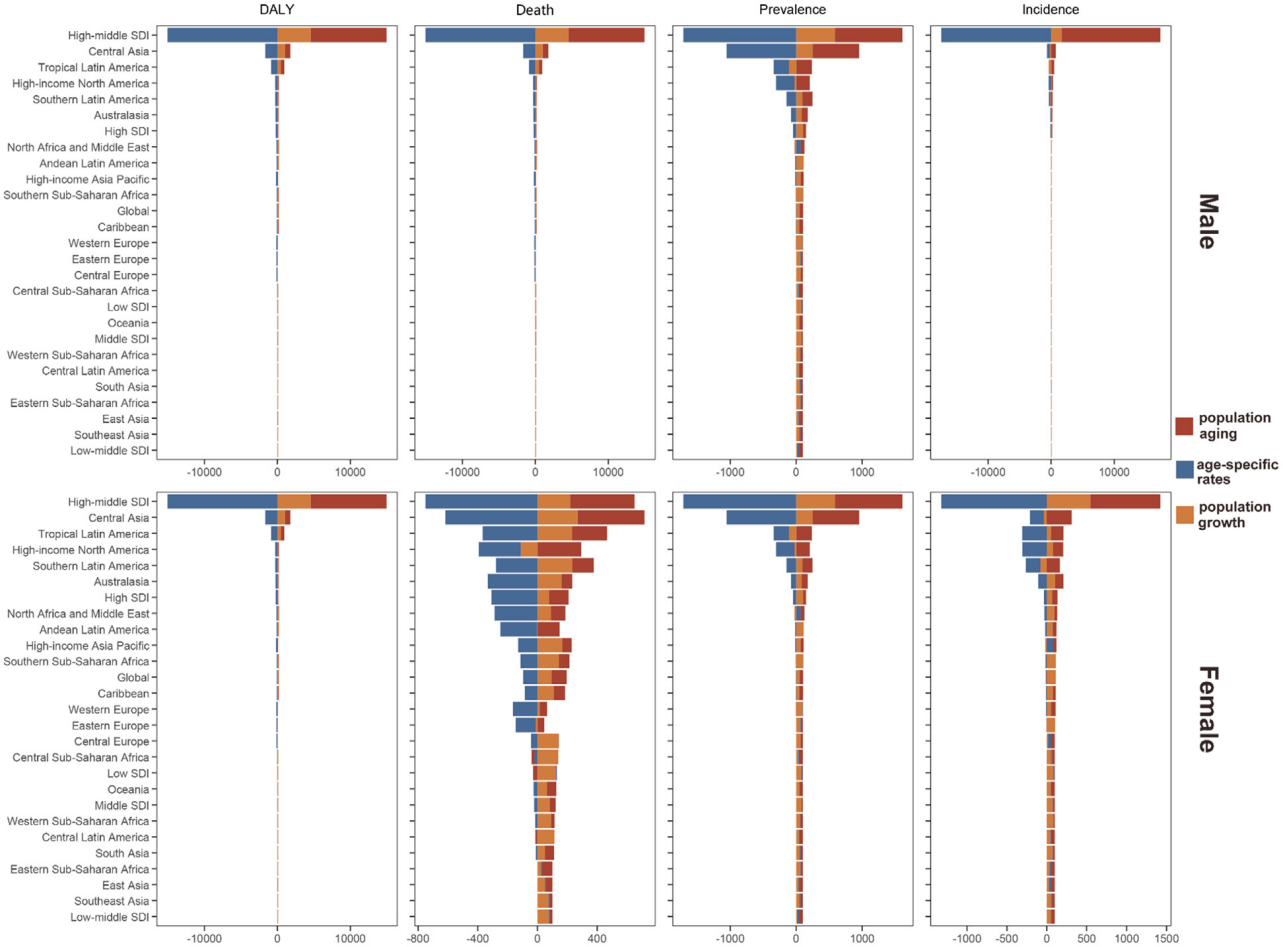
Decomposition Analysis of Changes in the Number of Age-Standardized Disability-Adjusted Life Year, Death, Prevalence, and lncidence Rate of Early-Onset Ischemic Heart Disease by Sex, Globally, in the Sociodemographic Index Region, and the 21GBD Region, From 1990 to 2019, as a Result of Population Growth, Population Aging, and Age-Specific Rates For each component, the magnitude of a positive value indicates a corresponding increase in the corresponding indicator of early-onset ischemic heart disease attributable to that component; the magnitude of a negative value indicates a corresponding decrease in the corresponding indicator of early-onset IHD for the relevant component. Abbreviations as in Figures 2 and 4.

From 1990 to 2019, ASIR, prevalence rate, DALY rate, and death rate for both males and females had a modest global impact, with more significant effects in high-middle SDI. These effects included increased incidence and prevalence due to population aging and age-specific changes. Despite this, there was a decrease in age-standardized DALY rate and death rate, attributed to the interplay between population aging, population growth, and age-specific rate change. In high-middle SDI, the rise in ASIR and prevalence rate among females was particularly pronounced, with notable impacts in countries such as Central Asia, Tropical Latin America, and South Latin America (Figure 5).

For both males and females, the changes in the number of incident cases, prevalence cases, DALY cases, and deaths were influenced by similar factors. Globally and in regions like South Asia and North America and Middle East, the increase in incident cases was largely driven by population aging, while the rise in deaths in middle-SDI, South Asia, North America and Middle East, and low-middle SDI was also primarily due to aging (Supplemental Figure 5).

## Discussion

### Major findings

According to Global Burden of Disease 2019, this study showed a significant increase in the number of early-onset IHD cases globally from 1990 to 2019, reaching 5.34 million cases. ASIR for both sexes shows a decreasing trend from 1990 to 2019. The top 3 attributable risk factors for men in 2019 were high LDL cholesterol, high systolic blood pressure, and smoking. In 2019, the top 3 attributable risk factors for men are high LDL cholesterol, high systolic blood pressure, and particulate matter pollution.

### Global incidence of early-onset ischemic heart disease

It is concerning to note that there has been a significant increase in global early-onset IHD cases, which is consistent with the observation of increased trends in premature coronary artery disease.^8^^,^^11^^,^^12^^,^^27^ The number of cases of early-onset IHD has risen from 1.51 million in 1990 to 2.57 million in 2019 for men and from 1.50 million in 2019 to 2.77 million in 2019 for women. Currently, the incidence of early-onset IHD is on the rise globally, with some studies suggesting that this trend is more pronounced in developing countries.^27^^,^^28^ However, it is worth mentioning that despite this alarming rise in cases, the trend in ASIR has continued to stabilize.^29^

The cause of this rise in cases of early-onset IHD may be linked to the rising prevalence of obesity, diabetes, and metabolic disease. Furthermore, changes in diagnostic methods may also have played a role in this disconcerting trend. Perhaps by understanding the temporal trends in early-onset IHD, we can gain valuable insights into the disease and develop informed public health strategies.

### Socio-demographic indices differences

The global prevalence of early-onset IHD is truly remarkable, and its incidence is largely influenced by changes in people's lifestyles due to environmental pollution and economic development.^30^ These changes are particularly evident in regions with low SDI, where epidemiological data have revealed that populations in North Africa and Central Asia experience a significantly higher ASIR compared to their higher-income counterparts in the Americas or Asia Pacific.^31^^,^^32^ Globally, myocardial ischemia and heart disease also show different patterns of prevalence and mortality. Prevalence is declining in high SDI countries and increasing in low SDI countries, and among mortality rates, IHD mortality has declined in high SDI countries, while it remains high in low and middle SDI countries.^14^ This may be related to factors such as socioeconomic status, level of education, access to health care, and health behaviors.^33^^,^^34^ Nevertheless, our study has revealed a puzzling finding: the prevalence and incidence of early-onset IHD remain numerically lower in low SDI countries compared with high SDI countries.^6^ This raises curiosity regarding the explanations behind such a result. Could this disparity be attributed to a greater number of medical visits among populations in high SDI countries, or perhaps the extended average life expectancy observed in these populations? The intricacies surrounding this disparity demand further investigation as they hold the potential to provide valuable insights for addressing this major global health issue.^35^

### Sex difference

A sex difference has been established in the incidence and burden of IHD. Previous studies conducted in China have unequivocally reported higher rates in men than in women.^36^^,^^37^ Similarly, alarming data from the United States and Asia suggest that men have double the burden of cardiovascular disease, including IHD, compared to women across all states.^38^, ^39^, ^40^ Echoing these findings, our study showed that during the period 1990 to 2019, ASIR, age-standardized DALY rate, and ASDR were higher in men than in women, although the number of incidence and prevalence rates were higher in women than in men. This takes into account the different ages selected for early-onset IHD in males and females.^35^ Despite these changes, ASIR was still higher in men than in women in low SDI and high-middle SDI countries. Also in our study, age-standardized DALY and ASDR were much higher in men than in women in the same SDI countries.^41^ The ASDR for early-onset IHD was also shown to be higher in males compared to females in a previous study, which is consistent with what we found.^31^^,^^38^ Truly, the intricate interplay between sex differences and early-onset IHD adds a layer of depth to this already complicated global health issue.

### Attributable risk factors for early onset IHD

Factors contributing to the early onset of myocardial infarction are multiple and complex, including biological and environmental factors.^42^ Atherosclerosis, an inflammatory arterial disease caused by lipid deposition and metabolic changes, is the main pathological process leading to IHD. Biological factors such as genetics, sex, high systolic blood pressure,^43^^,^^44^ high fasting glucose^15^^,^^45^^,^^46^ and age, as well as behavioral and environmental factors including high LDL cholesterol, smoking,^47^ and high body mass index,^48^ are all significant contributors to the development of IHD.^49^^,^^50^

While many of these factors have been discussed in previous research,^51^ gaps in IHD remain the risk factors in men and women.^32^ Our findings showed that high LDL-C was the number one ranked causative factor in both men and women, highlighting the need to control conventional risk factors in the prevention of early-onset IHD. In addition, particulate matter pollution ranked third in women and fourth in men, highlighting new evidence linking air pollution (eg, PM2.5 exposure) to cardiovascular disease.^43^^,^^52^

In addressing the complex challenges posed by this global health issue, developing national environmental health policies can provide a promising solution.^53^ These policies could help mitigate the impact of ambient PM2.5 exposure on the population, potentially reducing regional differences in IHD mortality rates. However, the causal mechanisms underlying the link between national policies and mortality from ambient PM2.5-induced IHD remain unclear and warrant further investigation.^31^

These results indicate that early intervention is crucial in mitigating the impact of these varied risk factors on the onset of IHD. It is imperative to develop targeted measures to prevent or reduce exposure to these risk factors and promote healthy behaviors to prevent the premature onset of this debilitating disease.^54^

### Study Strengths

Previous research has primarily focused on examining the prevalence and trends of IHD.^6^^,^^14^^,^^34^^,^^55^^,^^56^ Our study assessed the global burden of early-onset IHD in terms of global prevalence, incidence, DALY, trends in death by geographic location, SDI, age, and sex, as well as attributable risk factors over the period 1990 to 2019.

### Study Limitations

Since the GBD2019 incidence rates for IHD are confined to myocardial infarction, this study may underestimate the total early-onset IHD incidence, particularly in populations where non-myocardial infarction presentations are more prevalent.^3^ It is acknowledged that there are limitations that may contribute to statistical bias. The inclusion criteria for data collection in the GBD study may inherently introduce statistical bias due to variations in diagnostic methods, with limited access resulting in lower observed prevalence. The advancement of powerful diagnostic tools such as percutaneous coronary angiography, coronary computed tomography angiography, and cardiac-specific troponin tests has enhanced diagnostic certainty. Furthermore, the reliance on the effectiveness of vital registration systems across different countries is also a significant factor. Although in cases where data are absent, modeling is often utilized, it may potentially introduce biases into the results. Consequently, the incomplete data systems in low SDI countries might lead to an underestimation of the prevalence or incidence of conditions. Given these limitations, estimates must be reported with an UI, which should be carefully considered as it reflects both data sparsity and differences in sample size across various data sources and study locations.^57^ It is also unfortunate that this aspect of substance use was not addressed in the section on attributable risk. Despite these limitations, our findings fill a significant data gap in assessing the global burden of early-onset IHD and provide valuable information for the management of IHD.

### Comparison with previous studies

IHD is the leading cause of death and disability among the cardiovascular disease worldwide. While several studies reported the early-onset burden of cardiovascular diseases^21^^,^^58^ the systematic description of early-onset IHD has not been assessed. In addition, there are gaps in our knowledge of cardiovascular disease attributable to risk factors in early-onset IHD. Our findings provide important policy-relevant insights to help develop the preventive and therapeutic strategies necessary to address the increasing burden of acute myocardial infarction morbidity. This information is critical for the development of effective policies and interventions for the management of early-onset IHD.

## Conclusions

According to the Global Burden of Disease study, the ASIR of early-onset IHD has declined from 1990 to 2019. The overall age-standardized DALY rate and ASDR of early-onset IHD were higher in low-middle SDI countries compared with high SDI and high-middle SDI countries. Men had a higher age-standardized DALY rate and ASDR of early-onset IHD than women. The main risk factors for DALY were high LDL-C and high systolic blood pressure.

Perspective **COMPETENCY IN MEDICAL KNOWLEDGE:**

IHD is the most common type of heart disease. Recent evidence from epidemiological studies indicate a concerning increase in early-onset IHD among younger individuals. The global mortality rates and disability-adjusted life years of early-onset IHD decreased over the past three decades. Studies have shown a steady trend in the incidence and prevalence of early-onset IHD. Age-standardized incidence and death are higher in low-middle SDI countries and men. High LDL- cholesterol and high systolic blood pressure are major contributing factors.

**TRANSLATIONAL OUTLOOK 1:** The pronounced burden of early-onset IHD highlights the importance of identifying high-risk individuals early, as this could help reduce premature deaths and disability.

**TRANSLATIONAL OUTLOOK 2:** Regions with higher mortality burden, such as low-middle sociodemographic index countries, may require focused public health screenings and interventions. Tailored programs addressing major modifiable risk factors—high low-density lipoprotein cholesterol and elevated systolic blood pressure—may help alleviate the disproportionate burden in these areas.

## Funding support and author disclosures

This work was supported by the 10.13039/501100001809Natural Science Foundation of China (No. 82160371 to Dr Jing Zhang, No. 82100869 and No. 82360162 to Dr Peng Yu); 10.13039/501100003453Natural Science Foundation of Guangdong (202201011395 to Dr Xiao Liu); Basic and Applied Basic Research Project of Guangzhou (202201011395 to Dr Xiao Liu); Natural Science Foundation in Jiangxi Province grant [20224ACB216009 to Dr Jing Zhang]; the Jiangxi Province Thousands of Plans (No. jxsq2023201105 to Dr Peng Yu); and the Hengrui Diabetes Metabolism Research Fund (No. Z-2017-26-2202-4 to Dr Peng Yu). All other authors have reported that they have no relationships relevant to the contents of this paper to disclose.
