# Supplementary material for: Global Burden of Early-Onset Ischemic Heart Disease, 1990 to 2019
Source: JACC Adv. 2024 Dec 20;4(1):101466. doi: 10.1016/j.jacadv.2024.101466 (PMC11731480; doi:10.1016/j.jacadv.2024.101466)
Supplement: Supplemental material [file mmc1.docx]

**Supplemental Figure 1 | Temporal trend of age-standardized prevalence rate (per 100,000 persons) for the burden of early onset ischemic heart disease, globally and among different SDI quintiles from 1990 to 2019. SDI, Socio-Demographic Index.**


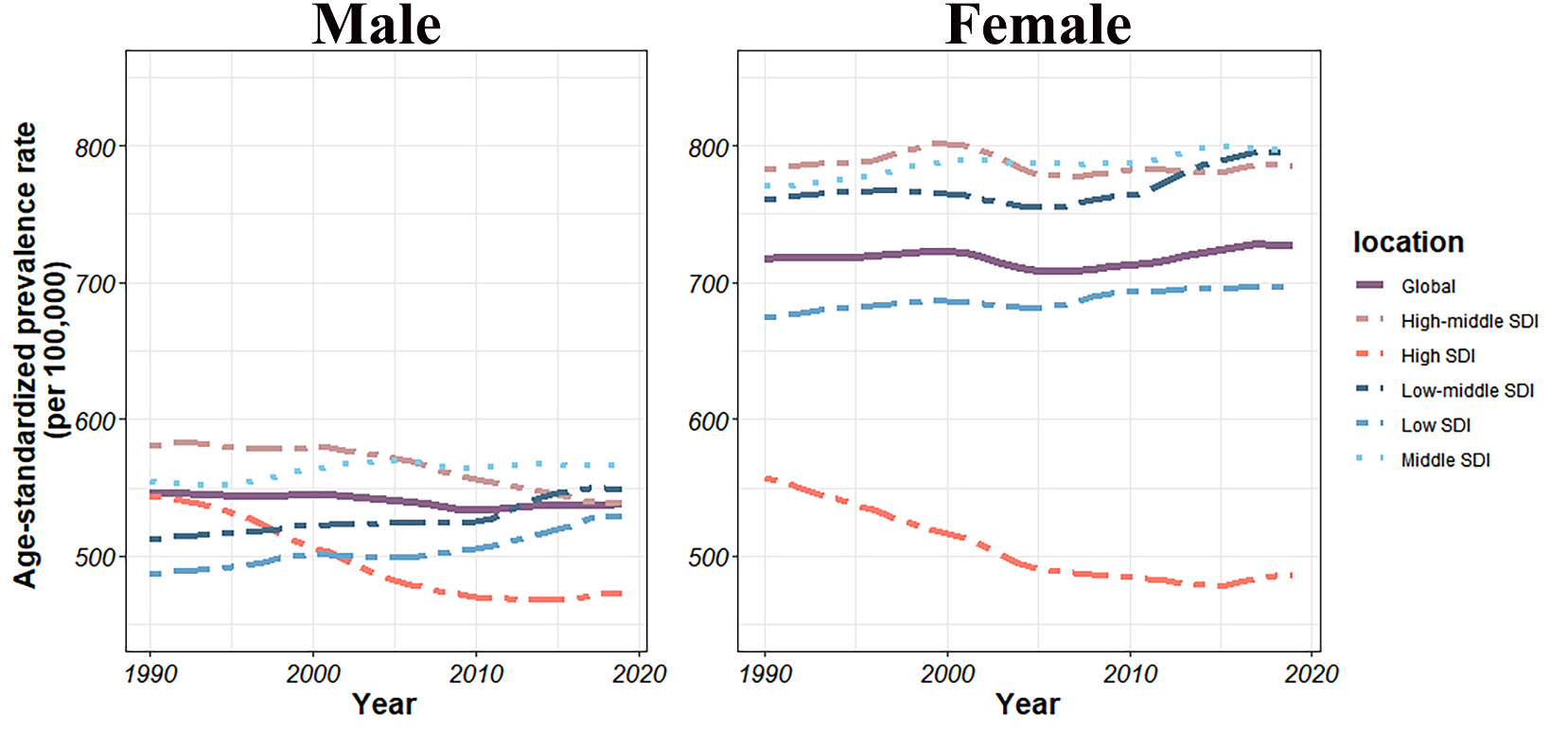


**Supplemental Figure 2 | Joint point regression analysis of sex-age standardized DALY ,death, incidence and prevalence rates in patients with ischemic heart disease worldwide, 1990 - 2019. (a) Age-standardized rate of DALY. (b) Age-standardized rate of death. (c) Age-standardized rate of incidence. (d) Age-standardized rate of prevalence. Squares: female standardized rates. Dots: male standardized rates.**

**
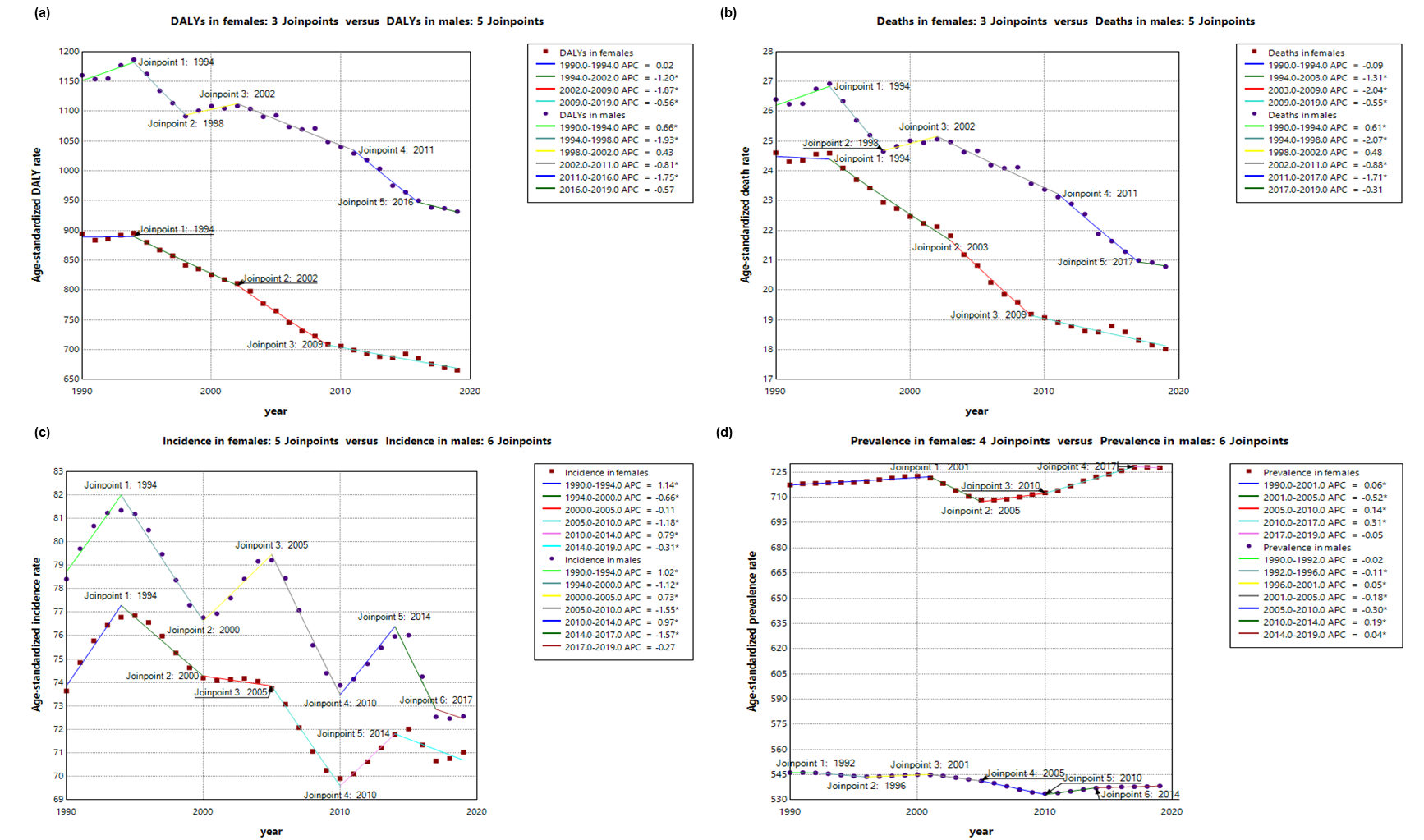
**

**Supplemental Figure 3 | Global age-specific incidence, prevalence, DALY rates and mortality in men and women, 1990-2019. (Males <55 years, females <65 years). DALY, disability-adjusted life year.**

**
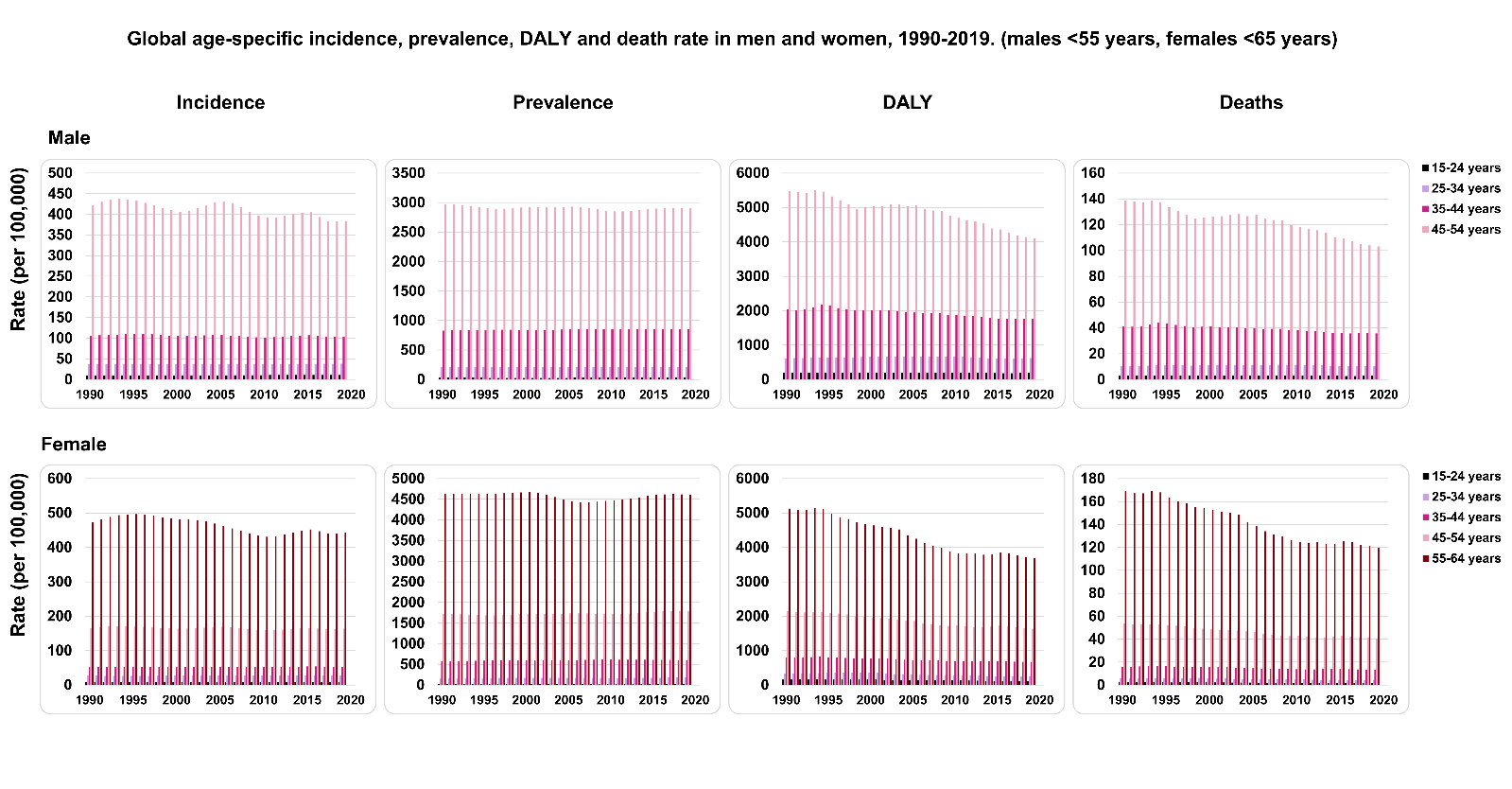
**

**Supplemental Figure 4 | Age-standardized DALY rate absolute and relative health inequality curves for ischemic heart disease and concentration curves for men and women, 1990-2019. (a) Age-standardized DALY rate absolute and relative health inequality curves and concentration curves for ischemic heart disease in men. (b) Age-standardized DALY rate absolute and relative health inequality curves and concentration curves for ischemic heart disease in women. DALY, disability-adjusted life year.**

**
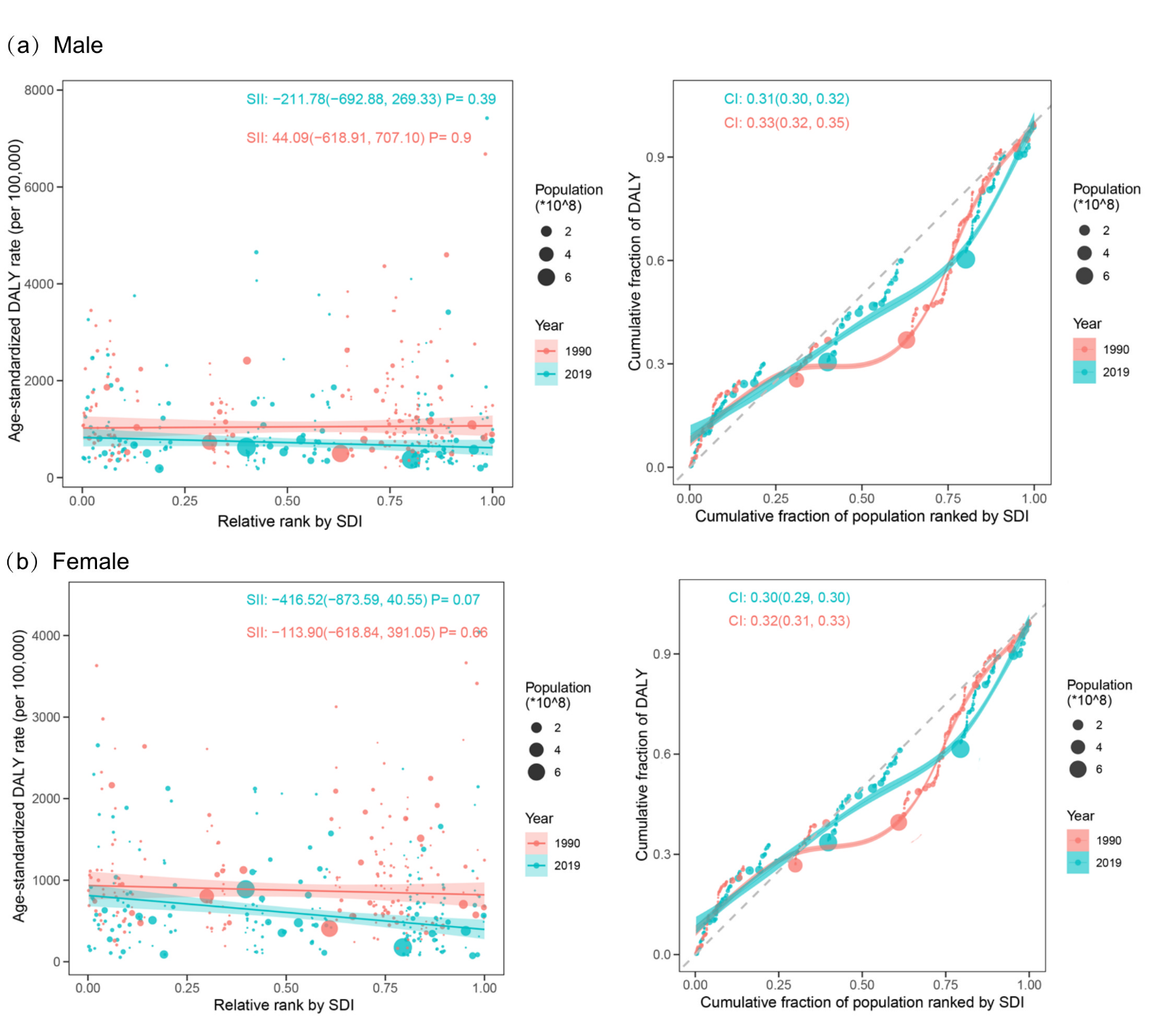
**

**Supplemental Figure 5: Decomposition analysis of changes in the number of cases of early-onset ischemic heart disease prevalence, incidence, DALY, and death by sex from 1990 to 2019, globally, in the SDI region, and in the 21GBD region, as a result of population growth, population aging, and age-specific rates. Black dots indicate the overall change in values contributed by population growth, population aging, and age-specific incidence rates. For each component, the magnitude of a positive value indicates a corresponding increase in the corresponding indicator of early-onset IHD attributable to that component; the magnitude of a negative value indicates a corresponding decrease in the corresponding indicator of early-onset IHD for the relevant component. DALY, disability-adjusted life year; SDI, Socio-Demographic Index.**

**
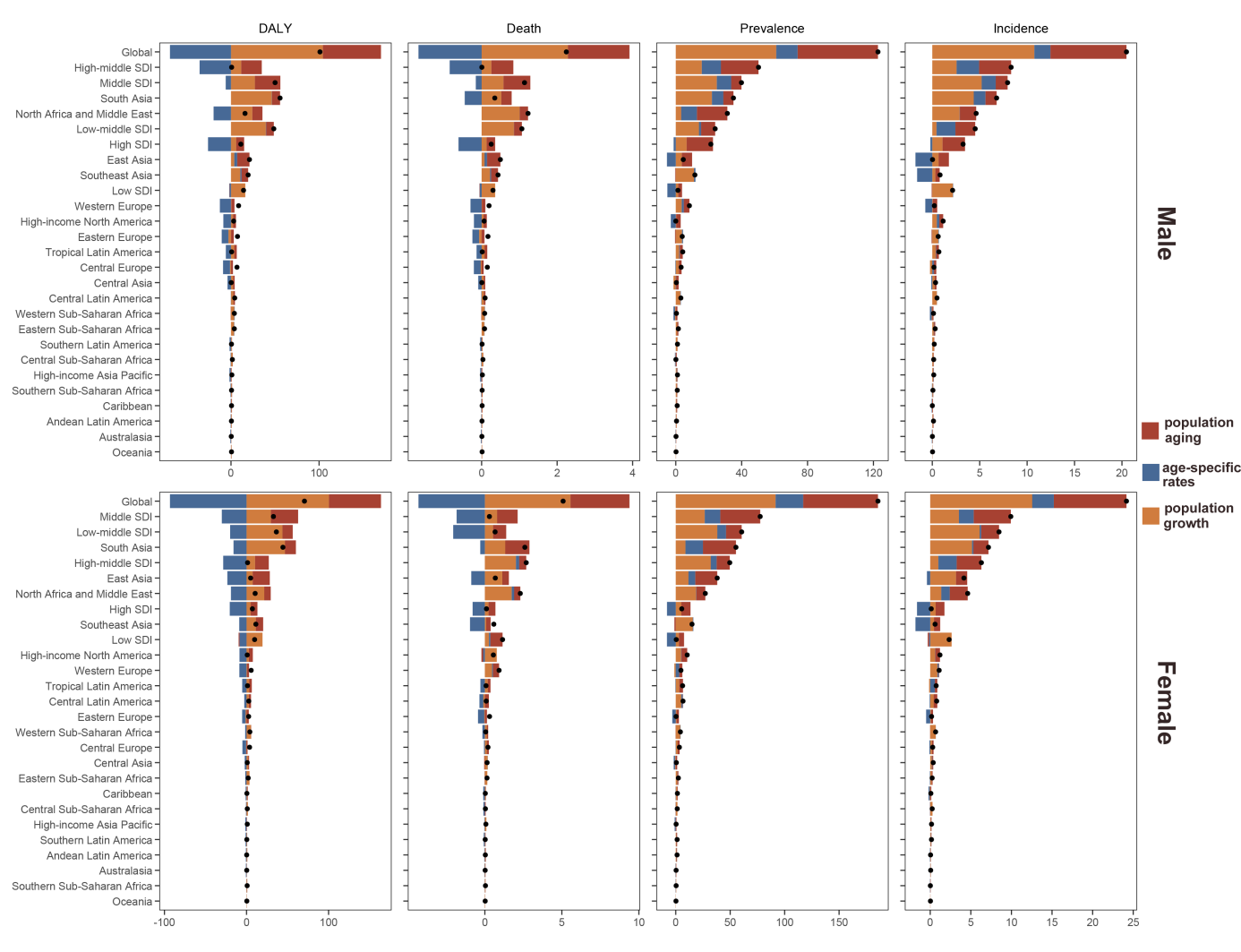
**
